# Rapid Aeolus L2B HLOS Wind Retrieval via BP Neural Network

**DOI:** 10.3390/s26041379

**Published:** 2026-02-22

**Authors:** Qinming Bi, Jiangang Lv, Pengfei He, Lusheng Zhang

**Affiliations:** 1 College of Information and Electrical Engineering, China Agricultural University (East Campus), Beijing 100083, China; 2023306100415@cau.edu.cn; 2 School of Physics and Electronic Information, Yantai University, Yantai 264005, China

**Keywords:** Aeolus satellite, Doppler wind lidar, BP neural network, HLOS

## Abstract

Wind field information is a key variable in atmospheric science and weather prediction, and spaceborne Doppler wind lidar provides unique global observations of the horizontal line-of-sight (HLOS) wind. This study develops a data-driven model that maps Aeolus Rayleigh-channel Level-1B (L1B) observables to the operational Level-2B (L2B) HLOS wind product. Using the two Rayleigh discriminator responses as inputs, we train a backpropagation (BP) neural network to learn the nonlinear relationship between Rayleigh-channel measurements and the collocated L2B HLOS winds. The proposed approach is intended as a computationally efficient emulation/approximation of the L2B HLOS output from L1B observations, rather than as an independently validated accuracy-improving retrieval. Model performance is evaluated by agreement with the L2B reference across samples spanning July 2019 to May 2020 and an altitude range of 0–20 km. The results show that the proposed model reproduces the main statistical characteristics and along-track HLOS patterns of the L2B product, providing a fast option for generating L2B-like HLOS estimates from Rayleigh-channel inputs.

## 1. Introduction

The atmospheric wind field is one of the most important physical parameters characterizing climate states and meteorological conditions [1]. Accurate observation of its temporal evolution and spatial distribution is essential for investigating atmospheric dynamics and thermodynamics, understanding transport and energy coupling, and improving numerical weather prediction. However, global wind-profile observations remain relatively sparse compared with other atmospheric variables, motivating the development of advanced wind-sensing techniques.

Wind field observation techniques can be broadly classified into ground-based, airborne, and spaceborne approaches. Among these, spaceborne wind sensing provides near-global coverage and consistent sampling, and can complement conventional observing systems. Passive approaches based on airglow emissions are effective in the upper atmosphere. However, they are limited in the lower atmosphere due to weak radiance, whereas active sensing (notably Doppler wind lidar) is well suited for retrieving winds in the troposphere and lower stratosphere.

To address the demand for low-altitude wind observations, the European Space Agency launched the Atmospheric Dynamics Mission Aeolus (ADM-Aeolus) satellite in 2018 [2]. Its payload, ALADIN, is a Doppler wind lidar that delivers operational HLOS wind profiles from near the surface up to approximately 30 km. In practice, Aeolus wind retrieval is challenging because measurement and processing performance depend on scene conditions and signal-to-noise regime, including variations in clouds and aerosols, heterogeneous backscatter, and instrument stability. Consequently, the operational processing chain relies on calibration, retrieval assumptions, and quality control (QC) logic, and the quality and availability of the retrieved winds may vary with altitude, latitude band, surface type (land/ocean), and QC screening.

In recent years, machine learning has demonstrated strong capability in learning nonlinear mappings from large geophysical and remote-sensing datasets. It has been increasingly applied to meteorological prediction and satellite data processing (e.g., [3,4,5,6,7,8]), motivating us to explore a supervised data-driven model for mapping Aeolus Rayleigh-channel L1B observables to the operational L2B HLOS product.

In many applications (e.g., quick-look monitoring, rapid offline reprocessing, and latency-sensitive downstream analyses), it is desirable to obtain L2B-like HLOS estimates directly from observable-level inputs with a simple and reproducible mapping. Motivated by this engineering need, we formulate the problem as a supervised regression task that maps Rayleigh-channel L1B discriminator responses ($R_{a}$, $R_{b}$) to the collocated operational L2B HLOS wind output at the range-bin level. In this paper, we develop a backpropagation neural network as a data-driven emulator/approximation of the operational L2B HLOS product. Model evaluation is performed as agreement with the L2B reference under the adopted protocol; thus, the reported metrics quantify product-to-product consistency rather than independently validated improvement in geophysical accuracy.

The main contributions of this work are: (i) an explicit separation of input observables (L1B-only Rayleigh discriminator responses) and the target product (L2B HLOS label), including leakage control and a structured feature inventory; (ii) an end-to-end emulator that produces L2B-like HLOS estimates from Rayleigh-channel inputs over July 2019–May 2020 within 0–20 km; and (iii) diagnostic evaluation of agreement with the operational product, providing a reproducible baseline for future studies that incorporate stricter generalization protocols and independent-reference validation.

## 2. Fundamental Principles

### 2.1. Classification of Spaceborne Wind Measurement Techniques: Passive and Active Sensing

Spaceborne wind measurement techniques can be further divided into passive and active sensing approaches. Passive methods primarily utilize airglow emissions from atmospheric atoms or molecules as target sources, and employ high-spectral-resolution optical interferometric techniques to measure Doppler frequency shifts in airglow spectral lines for wind speed retrieval. In 2019, NASA launched the Ionospheric Connection Explorer (ICON) satellite, equipped with the Michelson Interferometer for Global High-resolution Thermospheric Imaging (MIGHTI). This instrument retrieves wind profiles in the altitude range of 90–300 km by measuring Doppler shifts of atomic oxygen emissions at 557.7 nm and 630.0 nm, achieving retrieval accuracies of approximately 1.2–4.7 m/s [9]. However, due to the lack of airglow emissions in the lower atmosphere, passive sensing methods encounter significant difficulties in this region. Notably, the lower atmosphere below 30 km is more closely related to human activities and socio-economic processes.

For lower-atmospheric wind field measurements, active sensing techniques are typically adopted, with spaceborne Doppler wind lidar being a representative example. The fundamental principle of Doppler wind lidar is to emit high-frequency, stable, narrow-linewidth pulsed laser beams into the atmosphere and retrieve wind speed profiles by analyzing Doppler frequency shifts contained in Rayleigh or Mie backscattered signals. Aeolus/ALADIN measures only the LOS Doppler shift, and the operational product is the HLOS wind component. Full 2D/3D wind vectors cannot be obtained from a single-LOS measurement without additional assumptions or auxiliary data (e.g., multi-view geometry or model constraints). Therefore, this study strictly focuses on the retrieval/emulation of HLOS. In 2018, the European Space Agency (ESA) successfully launched the ADM-Aeolus satellite [2]. The satellite is equipped with the ALADIN [10], which employs independent Mie and Rayleigh detection channels to measure wind fields from the surface up to an altitude of approximately 30 km, with an overall retrieval error of about 2–3 m/s.

### 2.2. System Architecture of the Aeolus ALADIN Instrument

The ESA invested approximately USD 550 million over a development period of 16 years and successfully launched the ADM-Aeolus satellite in 2018. Aeolus is the first satellite to carry a high-spectral-resolution Doppler wind lidar (DWL) into space. Its single payload, the ALADIN, is an active remote sensing instrument operating with ultraviolet laser radiation. The ultraviolet wavelength was selected to enhance measurement sensitivity to molecular backscatter in the atmosphere. By measuring molecular and aerosol backscattered signals, ALADIN is capable of probing atmospheric layers above the planetary boundary layer, thereby providing valuable information in both aerosol-rich and aerosol-free regions.

The satellite is equipped with a 13 m wide gallium arsenide (GaAs) solar array, supplying an average power of approximately 1.4 kW to support onboard subsystems. In addition, ADM-Aeolus carries a large-aperture Cassegrain telescope. The satellite forms a single-aperture, confocal Cassegrain optical system, which maximizes the collection efficiency of atmospheric backscattered light.

ADM-Aeolus operates in a polar, sun-synchronous dawn–dusk orbit at an average altitude of approximately 407 km, completing one orbit every 92 min and 29 s. The cross-track separation between adjacent orbits is about 2500 km at the equator, resulting in an along-track spacing of approximately 370 km between consecutive wind measurement points. Since atmospheric air masses predominantly undergo horizontal motion, wind measurements require an off-nadir viewing geometry. Consequently, ADM-Aeolus adopts a viewing angle of 35° toward the Earth’s night side, perpendicular to the satellite velocity vector.

The single LOS atmospheric Doppler wind lidar onboard Aeolus, ALADIN, operates at a laser wavelength of 355 nm. To enable comprehensive and accurate detection of various atmospheric components under different conditions, ALADIN combines both Mie and Rayleigh scattering mechanisms. Wind measurements are obtained by analyzing Doppler frequency shifts in lidar echoes from aerosol particles (including cloud droplets) and atmospheric molecules. The aerosol (Mie scattering) channel employs a Fizeau interferometer for fringe imaging [11,12,13,14], which retrieves wind information from particle backscattered signals. In contrast, the molecular (Rayleigh scattering) channel uses a double-edge Fabry–Perot interferometer [15,16,17] to measure winds in the lower stratosphere and troposphere over low-aerosol or aerosol-free regions.

**Figure 1:**
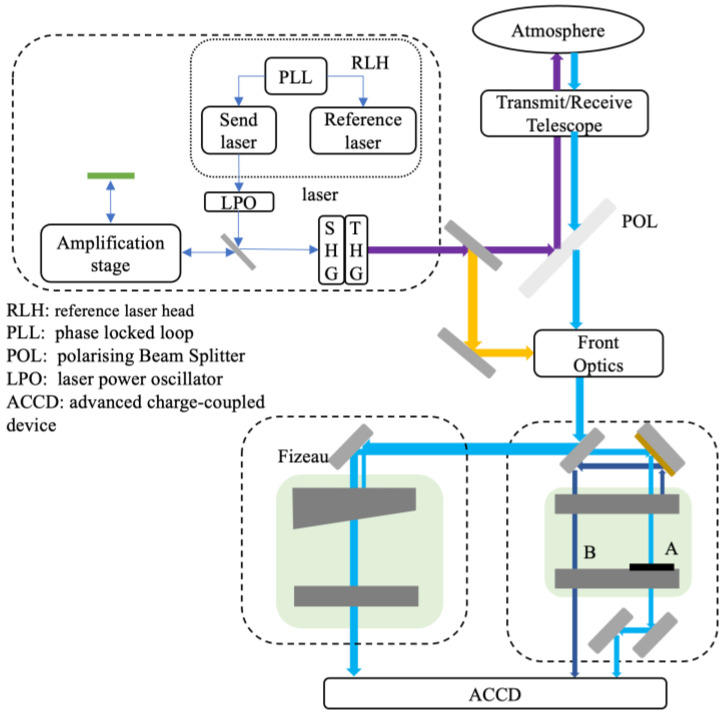
Optical Architecture of the Aeolus ALADIN System.

As illustrated in Figure 1, the ALADIN system consists of a laser transmitter, a telescope, and a receiver unit incorporating spectrometers. The laser transmitter adopts a master oscillator power amplifier (MOPA) configuration, which includes a seed laser and a reference laser. The reference laser system comprises two identical low-power lasers: one provides frequency stabilization, while the other enhances the energy of the transmitted laser pulses.

The emitted laser pulses are first frequency-doubled using nonlinear crystals and subsequently directed to a beam splitter. At the beam splitter, part of the optical signal is transmitted while the remainder is reflected. The reflected portion is used for wavelength monitoring, whereas the transmitted beam is further divided by another beam splitter into two paths: one for calibration and the other for atmospheric transmission. The telescope collects the atmospheric backscattered light and performs beam shaping through a front-end optical system. The beam is then directed by a polarization beam splitter toward the Mie spectrometer, with only a small fraction passing through the Fresnel aperture. Within the Fizeau interferometer, a wedge angle of 4.77 μrad between the spacer plates is introduced to control spectral dispersion, enabling the detection of narrowband particle backscatter signals (Mie channel). Approximately 90% of the optical intensity is reflected toward the Fabry–Perot interferometer to detect broadband molecular backscatter signals (Rayleigh channel). Finally, the detector unit collects photons transmitted through the spectrometers and outputs the measurement data to the data management system for subsequent processing and analysis.

### 2.3. Overview of Aeolus Satellite Data

The data used in this study are derived from publicly released product datasets of the ADM-Aeolus satellite operated by the ESA. To develop a data-driven wind field retrieval model, observations from the Rayleigh scattering channel of the ALADIN instrument are employed as the primary input features, while the corresponding HLOS wind speeds are adopted as the reference labels for supervised learning. The dataset provides global coverage (approximately 90°N–90°S) and enables wind field observations and retrievals from near the surface up to an altitude of about 30 km. To ensure data quality and reliability, quality flags included in the product files are utilized during the preprocessing stage to identify and remove abnormal or invalid records.

The Aeolus data processing chain follows the standard ESA product hierarchy, which includes the instrument source packet (AISP) as well as multiple processing levels, namely L0, L1A, L1B, L2A, and L2B. Among these, the L1B products provide geometrically located and radiometrically/instrument-calibrated channel observations together with auxiliary information, such as calibration files and quality flags, making them suitable as inputs for machine-learning models. In contrast, the L2B products deliver user-oriented HLOS wind products, which are directly applicable to meteorological analysis and numerical weather prediction applications.

### 2.4. Fundamental Principles of Wind Measurement Using the Aeolus Satellite

The atmosphere is primarily composed of gas molecules and may also contain aerosol particles (including cloud droplets), depending on scene conditions [18] and atmospheric molecules, which exhibit markedly different scattering characteristics. Owing to their much smaller size, atmospheric molecules predominantly produce Rayleigh scattering, whereas larger aerosol particles mainly generate Mie scattering. Due to differences in scattering cross sections, echoes produced by Mie scattering are generally stronger than those arising from Rayleigh scattering. In the lower atmosphere, Mie scattering is the dominant scattering mechanism, and its contribution exceeds that of Rayleigh scattering. However, in the altitude range of approximately 20–30 km, where aerosol concentrations are extremely low, the backscattered signal is primarily composed of Rayleigh scattering.

The Rayleigh channel is sensitive to molecular backscatter and is generally applicable across a broad altitude range in aerosol-poor conditions, whereas the Mie channel mainly provides information when particle backscatter is sufficiently strong (e.g., clouds/aerosols).

The ALADIN lidar system retrieves wind speed and direction information by transmitting ultraviolet laser pulses into the atmosphere and measuring the Doppler frequency shifts of the backscattered signals returned from atmospheric particles. In the presence of wind, the backscattered signals from both aerosol particles and atmospheric molecules experience Doppler shifts due to the relative motion between the scatterers and the lidar system. The Doppler frequency shift arises from the relative motion between the transmitted laser beam and the moving scatterers, causing the frequency of the received echo signal to vary with the velocity magnitude and direction of the scatterers. For molecular (Rayleigh) backscatter, the received spectrum is broadened by thermal (Brownian) motion of molecules, while the bulk wind induces a Doppler shift of the spectral centroid. Operational retrieval, therefore, estimates the LOS wind from averaged/filtered spectral information (e.g., discriminator responses), and the achievable accuracy depends on SNR and scene conditions.

The Doppler frequency shift induced by atmospheric motion is related to the line-of-sight wind velocity through the following expression:

(1) $$\Delta f=\frac{2V_{\mathrm{LOS}}}{\lambda },$$

where Δf denotes the Doppler frequency shift, *λ* is the wavelength of the transmitted lidar laser, and $V_{\mathrm{LOS}}$ represents the wind velocity component along the lidar line of sight. For a fixed lidar wavelength, a larger Doppler frequency shift corresponds to a higher line-of-sight wind velocity. Therefore, the LOS wind speed can be directly derived by accurately measuring the Doppler frequency shift.

**Figure 2:**
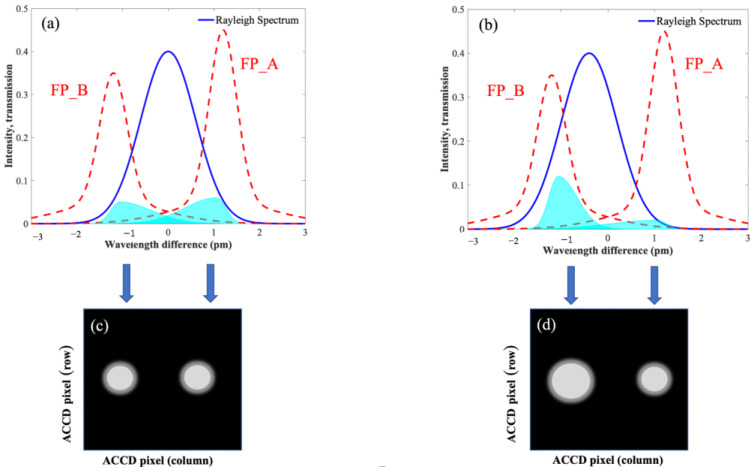
Schematic Diagram of the Rayleigh-Channel Wind Measurement Principle. (**a**) Transmission function and transmitted signal intensity of the FP_A channel; (**b**) Transmission function and transmitted signal intensity of the FP_B channel; (**c**) Simulated spot size on the ACCD imaging area under zero-wind conditions; (**d**) Simulated spot size on the ACCD imaging area under non-zero wind conditions.

As shown in Figure 2, the red dashed curves in panels (a) and (b) represent the transmission functions of the Fabry–Perot A (FP_A) and Fabry–Perot B (FP_B) channels, respectively, while the light-blue shaded regions denote the transmitted signal intensity after passing through the frequency discriminator. Here, “pm” denotes picometer (1 pm = 10^−12^ m) and is used to express the wavelength difference (Δλ) associated with the Doppler-induced spectral shift. Panel (c) illustrates the simulated spot size on the ACCD imaging area in the absence of wind, whereas panel (d) shows the simulated spot size under non-zero wind conditions. It can be observed that wind-induced variations in the echo frequency lead to changes in the signal intensities transmitted through the two channels, resulting in different spot sizes on the ACCD imaging area. By computing the number of detected photoelectrons in the FP_A and FP_B channels on the ACCD, both the wind speed and wind direction can be retrieved.

**Figure 3:**
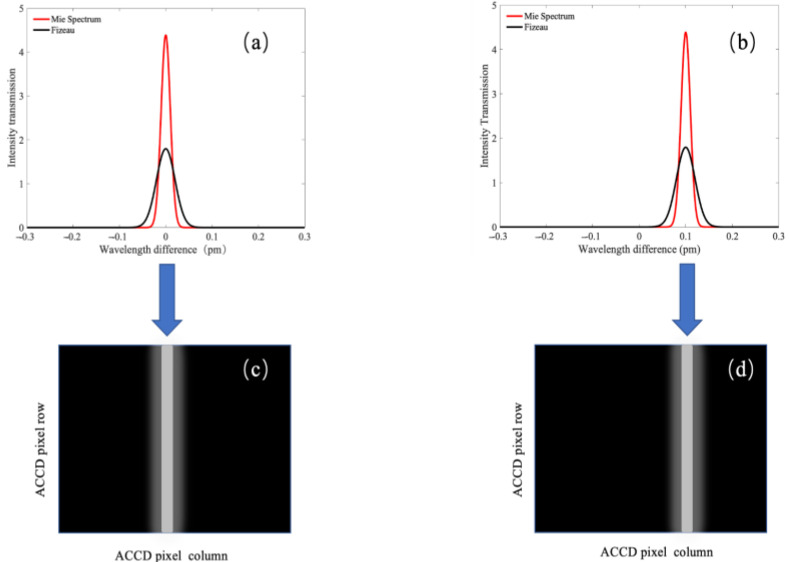
Schematic Diagram of the Mie-Channel Wind Measurement Principle. (**a**) Spectrum of the Mie-scattered echo signal and transmission function of the Fizeau interferometer; (**b**) Spectrum of the Mie-scattered echo signal and transmission function of the Fizeau interferometer under corresponding conditions; (**c**) Simulated spot position on the ACCD imaging area under zero-wind conditions; (**d**) Spectral variation of the echo signal induced by wind velocity changes.

As shown in Figure 3, the red solid curves in panels (a) and (b) represent the spectra of the Mie-scattered echo signals, while the black solid curves denote the transmission functions of the Fizeau interferometer. Panel (c) illustrates the simulated spot position on the ACCD imaging area under zero-wind conditions, whereas panel (d) shows the spectral variation of the echo signal induced by changes in wind velocity. By calculating the spectral shift of the backscattered echo signals, the wind speed and wind direction can be subsequently retrieved.

### 2.5. State-of-the-Art in Wind Field Retrieval Methods for ADM-Aeolus

Currently, wind retrieval methods for ADM-Aeolus data can be broadly classified into empirical retrieval approaches and physical-model-based retrieval approaches [19,20,21]. In terms of empirical retrieval, the German Aerospace Center (DLR) conducted airborne Doppler wind lidar campaigns in 2019 to calibrate the ALADIN instrument, achieving wind speed retrievals over land in the altitude range of 0–8.4 km with an error of approximately 3.9 m/s [20]. In the same year, Holger Baars et al. performed calibration over the Atlantic Ocean using radiosondes launched from aircraft, enabling wind retrievals over marine regions from 0 to 20 km with an error of about 3.3 m/s [20]. Regarding physical-model-based approaches, Rennie et al. applied the wind retrieval algorithm described in the L2B Algorithm Theoretical Basis Document in 2020 to retrieve horizontal line-of-sight wind speeds from ALADIN measurements, achieving errors of approximately 4–7 m/s, and further assessed the impact of these data on global numerical weather prediction systems [21]. In 2022, Oliver Lux et al. optimized the retrieval algorithm to obtain more accurate wind speed estimates from raw signals, reducing the error to approximately 1.5–3 m/s [22]. Nevertheless, the accuracy of horizontal line-of-sight wind speed estimates exhibits significant variability due to factors such as geographical location, season, software version, processor configuration, measurement range settings, and instrument performance. As a result, both empirical and physical-model-based retrieval methods face challenges in consistently guaranteeing retrieval accuracy and operational efficiency.

## 3. Model Training Methodology

Machine learning establishes mappings between input and output variables in a data-driven manner and is capable of effectively capturing nonlinear characteristics in complex systems, thereby offering significant advantages for remote sensing retrieval problems. To address challenges in spaceborne Doppler wind lidar wind field retrieval, including strong noise interference, complex physical models, and high computational costs, this study introduces a machine-learning approach to model the relationship between Rayleigh-channel observations from the ADM-Aeolus satellite and the corresponding HLOS wind speeds, enabling rapid retrieval of wind profiles.

### 3.1. Algorithm Description

In this study, the wind field retrieval problem is formulated as a supervised learning regression task. Specifically, observations from the Rayleigh scattering channel of the Aeolus satellite are used as input feature vectors, while the corresponding HLOS wind speeds serve as regression targets. A machine-learning model is trained to learn the nonlinear mapping between the Rayleigh-channel measurements and the HLOS wind speeds. Unlike traditional physics-based retrieval approaches, this method does not require the explicit construction of mathematical or physical models describing the relationship between scattering signals and wind velocity. Instead, it leverages large volumes of training samples to automatically extract underlying patterns, thereby improving retrieval efficiency and robustness.

During the model training process, each sample consists of the responses from the two frequency discriminators of the Rayleigh channel, together with relevant auxiliary information, which are used as input features, while the output is the HLOS wind speed at the corresponding altitude level. The objective of the model is to minimize the error between the predicted wind speeds and the reference wind speeds, enabling a computationally efficient emulation/approximation of the operational L2B HLOS winds under varying temporal, vertical, and geographical conditions. Feature selection rationale: To avoid circularity and ensure reproducibility, we restrict inputs to Rayleigh-channel L1B discriminator responses only (two responses), which are observable-level quantities directly related to Doppler-induced spectral asymmetry used in the operational processing. No L2B-derived variables, QC flags, or error estimates are used as inputs. This minimal input set provides a transparent baseline for emulator-style mapping.

Measurement scope and terminology. The ADM-Aeolus/ALADIN instrument provides horizontal line-of-sight (HLOS) winds, i.e., the projection of the horizontal wind vector onto the instrument line of sight. Under a single-LOS viewing geometry, the full horizontal wind vector and meteorological wind direction cannot be uniquely determined without additional assumptions and/or auxiliary information (e.g., multi-azimuth observations or model background winds). Therefore, this study strictly targets HLOS emulation/approximation: the inputs are limited to Rayleigh-channel L1B observables, optionally accompanied only by observation-geometry descriptors that do not encode L2B retrieval outcomes, and the target is the collocated operational L2B HLOS wind. Throughout the manuscript, any “direction” shown in figures refers to the LOS viewing azimuth/geometry or the sign of the HLOS component, rather than a retrieved 2-D wind direction.

### 3.2. Input Observables and Target Products (Leakage Control)

To minimize the risk of product-level information leakage, we explicitly separate input observables from the target product used for supervision. Inputs (L1B-only): Model inputs are restricted to observable-level quantities available in the ADM-Aeolus L1B products. In particular, the Rayleigh-channel inputs are the two discriminator responses, $R_{a}$ and $R_{b}$, from the two Rayleigh detection channels at each range bin. Any auxiliary variables included as inputs are limited to observation geometry descriptors and range-bin indexing information, and do not encode L2B retrieval outcomes. Target (L2B label): The supervision label is the ADM-Aeolus L2B HLOS wind at the same range bin, produced by the operational ESA processor. Leakage control: QC/validity flags are used exclusively for sample screening and are never fed into the network as input features. No L2B-derived fields or processor-coupled intermediate retrieval quantities are used as inputs. All normalization parameters are fitted on the training subset only and then applied to validation/test subsets to avoid leakage of test statistics. Feature inventory: A structured inventory of all input variables (name, unit, provenance, and preprocessing) is provided in Table 1.

### 3.3. Backpropagation Neural Network

The BP neural network [23], also referred to as the backpropagation neural network, is a widely used multilayer feedforward neural network that has been extensively applied to nonlinear regression problems. Its core principle lies in iteratively updating network weights and bias parameters through the backpropagation algorithm, with the objective of minimizing the error between model predictions and reference values. Given the pronounced nonlinear relationship between Rayleigh-channel observations and HLOS wind speeds, a BP neural network is adopted in this study as the wind profile retrieval model.

In this work, the BP neural network is employed to perform a supervised regression task. The network is trained by minimizing the mean squared error between the predicted wind speeds and the reference wind speeds, and a linear activation function is applied at the output layer to ensure appropriate continuous-valued outputs.

The weight updates of the BP network rely on the backpropagation algorithm [24], which propagates the error backward from the output layer and iteratively adjusts the network weights layer by layer to optimize overall network performance.

#### 3.3.1. Network Architecture and Parameter Initialization

In this study, a three-layer BP neural network architecture is adopted, consisting of an input layer, a hidden layer, and an output layer. The number of input neurons is determined by the dimensionality of the Rayleigh-channel feature vector, while the output layer contains a single neuron corresponding to the retrieved HLOS wind speed. The number of neurons in the hidden layer is determined through experimental comparisons.

During the initialization stage, the network weights $W^{\left[l\right]}$ are randomly initialized to avoid excessive sensitivity to the initial state, whereas the bias terms $b^{\left[l\right]}$ are initialized to zero. The weights and biases jointly determine the weighted inputs and activation states of neurons, thereby influencing the nonlinear representation capability of the network.

#### 3.3.2. Forward Propagation and Loss Function

During the forward propagation process, the weighted input and output of the neurons in the *l*-th layer are expressed as follows:

(2) $$z^{\left[l\right]}=W^{\left[l\right]}a^{\left[l-1\right]}+b^{\left[l\right]},$$

(3) $$a^{\left[l\right]}=g^{\left[l\right]}\left(z^{\left[l\right]}\right),$$

where $a^{\left[l\right]}$ denotes the output of the *l*-th layer, $W^{\left[l\right]}$ and $b^{\left[l\right]}$ represent the weight matrix and bias vector of the *l*-th layer, respectively, and $g^{\left[l\right]}\left(\cdot \right)$ is the activation function. Nonlinear activation functions (e.g., tansig or logsig) are employed in the hidden layer, while a linear activation function (purelin) is adopted in the output layer to accommodate the continuous-valued wind speed regression task.

The network is trained by minimizing the mean squared error (MSE) loss function, defined as follows:

(4) $$L=\frac{1}{N}\sum_{i=1}^{N}{\left(y_{i}-{\hat{y}}_{i}\right)}^{2},$$

where $y_{i}$ denotes the reference HLOS wind speed, ${\hat{y}}_{i}$ represents the corresponding model prediction, and N is the total number of training samples.

#### 3.3.3. Backpropagation and Parameter Update

During the backpropagation stage, the gradients of the loss function with respect to the network parameters are computed using the chain rule, and the weights and biases are updated via gradient descent as follows:

(5) $$w^{\left[l\right]}=w^{\left[l\right]}-\alpha \frac{\partial L}{\partial w^{\left[l\right]}},$$

(6) $$b^{\left[l\right]}=b^{\left[l\right]}-\alpha \frac{\partial L}{\partial b^{\left[l\right]}},$$

where α denotes the learning rate, which controls the step size of parameter updates. An appropriate choice of the learning rate helps accelerate the training process while ensuring the convergence stability of the model.

### 3.4. Data Preprocessing

The data used in this study were obtained from publicly released ADM-Aeolus products provided by the European Space Agency (ESA). We use observations covering the period from July 2019 to May 2020. The model inputs are extracted from the Rayleigh-channel L1B product (two discriminator responses, $R_{a}$ and $R_{b}$) (Section 3.2 and Table 1), and the supervision labels are the collocated L2B HLOS winds. The retrieval is evaluated within an altitude range of 0–20 km at the range-bin level.

**Figure 4:**
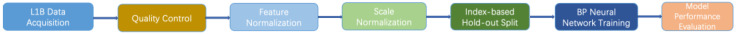
ADM-Aeolus Data Preprocessing Flowchart.

Quality control (QC) and effective sample size. As shown in Figure 4, QC is first applied to ensure the reliability of training data. During data screening, abnormal records are removed using the validity/quality information provided in the product files. Specifically, samples with non-zero validity flags are regarded as invalid and discarded. In addition, samples with missing/NaN values in either the inputs ($R_{a}$, $R_{b}$) or the target HLOS wind are removed. After QC, the effective dataset contains approximately $N_{samples}$ = 1 × 10^4^ bin-level samples within 0–20 km.

Signal normalization. After removing abnormal samples, normalization is applied to the input features to mitigate amplitude variations caused by factors such as laser energy fluctuations and changes in instrument response. This preprocessing step improves numerical stability and accelerates convergence during model training. The normalization procedure is described as follows:

(7) $$X^{\prime }=\frac{R_{a}/{R^{\prime }}_{a}}{R_{b}/{R^{\prime }}_{b}},$$

where ${R^{\prime }}_{a}$ and ${R^{\prime }}_{b}$ denote the mean values of the reference signals from channels a and *b*, respectively. This normalization strategy helps standardize the input data, enabling a more accurate representation of the atmospheric backscattered signal intensity.

Train/test split and normalization consistency. For evaluation, the collocated samples are split into training and test subsets by index (i.e., the first portion for training and the remaining portion for testing; approximately [80%/20%]). All scaling parameters (e.g., min–max scaling) are fitted on the training subset only and then applied to the test subset using fixed parameters, avoiding any leakage of test statistics into training. We note that Aeolus observations exhibit spatial–temporal autocorrelation along orbits; therefore, index-based or sample-level splitting may yield optimistic estimates of out-of-block generalization. This limitation is explicitly acknowledged, and stricter block-by-time/orbit holdouts are left for future work.

Model setting selection. The main network settings (e.g., number of hidden neurons and training parameters) are chosen based on validation performance within the training subset, balancing retrieval accuracy and computational cost.

Evaluation metrics. To quantitatively evaluate the retrieval performance of the proposed model, the mean squared error (MSE), root mean squared error (RMSE), mean absolute error (MAE), and coefficient of determination ($R^{2}$) are employed as evaluation metrics. These metrics characterize the discrepancies between the predicted wind speeds and the reference wind speeds from different perspectives, enabling a comprehensive assessment of model accuracy and reliability.

### 3.5. Training of the BP Neural Network Model

In this study, a BP neural network–based model is designed for wind profile retrieval using data from the Aeolus satellite. The BP neural network consists of an input layer, a hidden layer, and an output layer. Considering that the number of input features n includes wind profile–related parameters and Rayleigh-channel observations, and that the number of output labels 1 corresponds to the retrieved wind profile values, a three-layer network architecture (one input layer, one hidden layer, and one output layer) is adopted. In the training process, the hidden layer is configured with 20 neurons to construct the BP neural network model.

As indicated by the results summarized in Table 2, the model exhibits superior performance when the sigmoid activation function is employed in the hidden layer, and the purelin activation function is used in the output layer. This configuration not only achieves lower prediction errors but also provides stronger explanatory capability with respect to the underlying data.

In the training of the BP neural network, the selection of appropriate termination criteria is crucial. Common stopping conditions include reaching a predefined error threshold or completing a specified number of training iterations. In addition, the choice of a suitable learning rate is essential to avoid excessive oscillations near the optimal solution, which may hinder convergence. Based on empirical experience, the training process in this study is configured with a maximum of 2000 training iterations, a learning rate of 0.005, and a target training error of 0.00001, thereby ensuring both stability and efficiency during model training.

## 4. Comparison and Analysis of Model Retrieval Performance

After systematically evaluating the retrieval performance of the BP neural network model under different parameter configurations, this study further analyzes the prediction errors produced by the model. Through quantitative statistics and diagnostic plots, we assess the distribution characteristics of residuals and identify potential limiting factors under practical application settings.

In Table 3, bias is defined as mean(pred − L2B), and all metrics are computed in physical units (m/s) after inverse normalization.

### 4.1. Comparison of Wind Field Vectors

**Figure 5:**
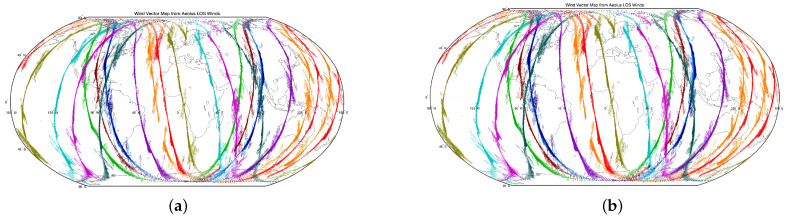
Along-track HLOS wind comparison (L2B reference vs. BP estimate). (**a**) Operational Aeolus L2B HLOS wind (reference); (**b**) BP-model HLOS estimates from Rayleigh-channel L1B discriminator responses.

In this subsection, we compare the operational Aeolus Level-2B (L2B) horizontal line-of-sight (HLOS) wind product with the BP-model estimates obtained from Rayleigh-channel Level-1B (L1B) observables, in order to assess product-to-product agreement and characterize the associated errors. Figure 5 shows the along-track spatial distribution of HLOS winds during the study period.

Figure 5a shows the collocated L2B HLOS wind values along the satellite tracks, which serve as the reference in this study. Figure 5b shows the corresponding BP-model HLOS estimates generated from the Rayleigh-channel discriminator responses. The comparison illustrates that the BP model can reproduce the main large-scale along-track HLOS patterns of the operational L2B product. Independent validation against external references is left for future work.

By analyzing the differences in vector directions and magnitudes between the two panels, systematic biases or random errors in the model performance can be identified in specific regions.

**Figure 6:**
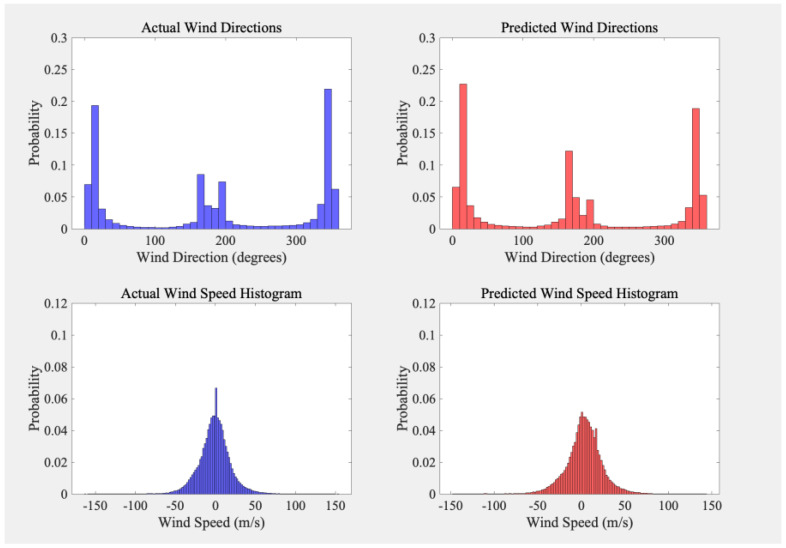
Histograms of Observed and Predicted Wind Speed and Direction.

Figure 6 compares the distributions of the LOS viewing azimuth angle and HLOS wind speed for the collocated and QC-filtered samples. The upper panels show the histogram of the LOS viewing azimuth angle (degrees) derived from the Aeolus observation geometry. This variable reflects the LOS viewing direction rather than the true horizontal wind direction, since only the HLOS component is available in the L2B product. The lower panels show histograms of HLOS wind speed (m/s) for the reference and model results, which help assess whether the emulator reproduces the main statistical characteristics of the operational L2B product.

**Figure 7:**
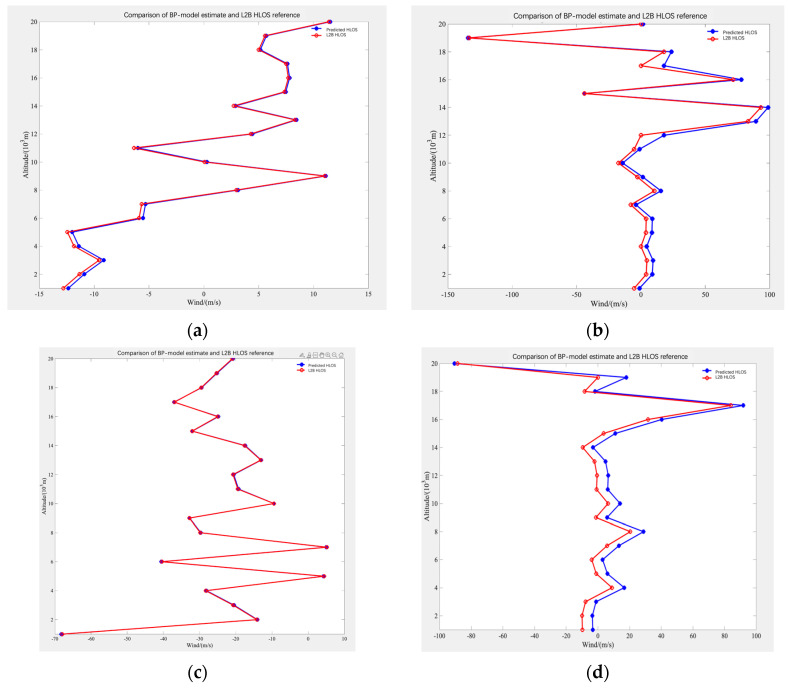
Comparison of Observed and Predicted Wind Speed. (**a**) Southwestern Hemisphere; (**b**) Northwestern Hemisphere; (**c**) Southeastern Hemisphere; (**d**) Northeastern Hemisphere.

Figure 7 presents a comparison of wind speeds at different altitudes between model predictions and actual observations across different regions from July 2019 to May 2020. Panels (a), (b), (c), and (d) correspond to the Southwestern Hemisphere, Northwestern Hemisphere, Southeastern Hemisphere, and Northeastern Hemisphere, respectively.

**Figure 8:**
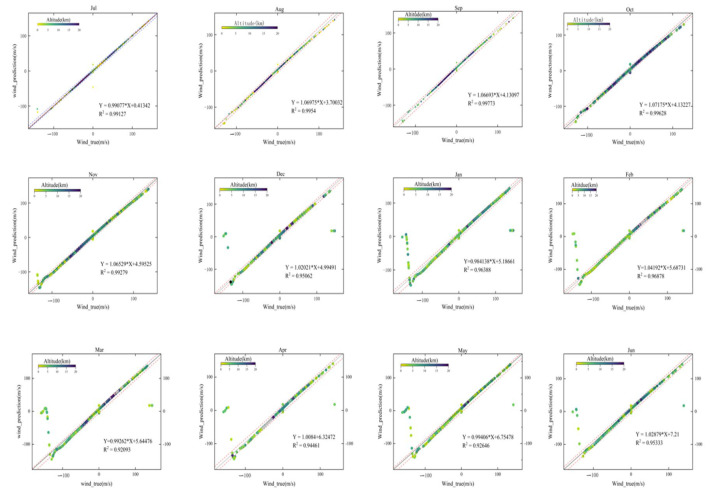
Linear Regression Plot.

Figure 8 shows the relationship between predicted and observed wind speeds using a scatter plot, with data points color-coded according to altitude.

Through systematic comparison with the operational L2B HLOS reference product, the results demonstrate a high degree of agreement between the BP-model estimates and the L2B winds. The predicted HLOS profiles reproduce the overall vertical structures and numerical distributions of the collocated L2B profiles from near the surface up to approximately 20 km. In particular, close agreement is observed at representative altitude levels (e.g., around 6 km, 12 km, and 16 km) in the examples shown. These findings indicate that the proposed model can emulate/approximate the L2B HLOS output from Rayleigh-channel L1B inputs. Independent validation against external references (e.g., radiosondes or ECMWF analyses) is left for future work.

**Figure 9:**
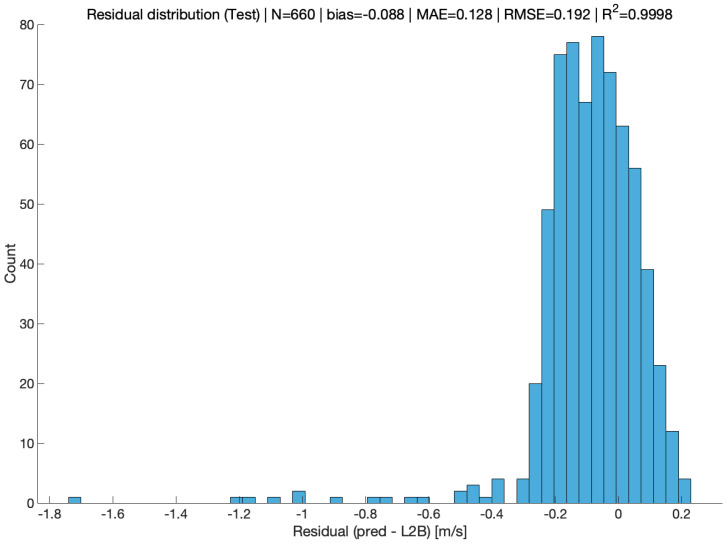
Residual distribution on the test set (pred − L2B, in m/s).

Through systematic comparison with the operational L2B HLOS reference product, the results demonstrate a high degree of agreement between the BP-model estimates and the L2B winds. Figure 8 shows the overall correspondence between predicted and reference HLOS winds. To provide a more direct error-level diagnostic beyond correlation plots, Figure 9 presents the distribution of test-set residuals (pred − L2B), including summary statistics (Bias, MAE, RMSE, and *R*^2^).

### 4.2. Comparison of Wind Profiles

To evaluate the capability of the model to capture the vertical structure of wind speed, the wind profiles predicted by the model trained on the training dataset are compared with the corresponding observed wind profiles. The vertical distribution characteristics of the wind profiles provide an intuitive assessment of the model’s ability to reproduce wind speed variations across different altitude levels.

**Figure 10:**
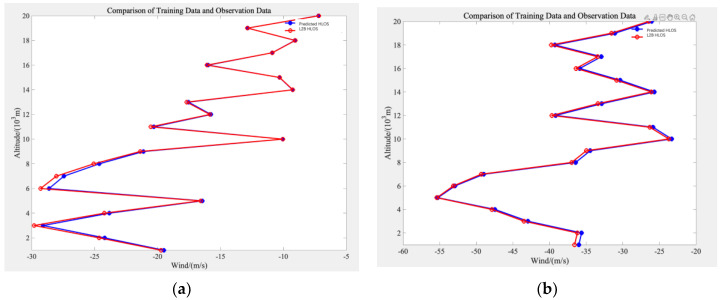
Comparison of Training-Set Wind Profiles and Observed Wind Profiles. (**a**) Wind speed profile comparison at Time 1; (**b**) Wind speed profile comparison at Time 2.

Figure 10 presents a comparison between the wind speed profiles predicted by the model on the training dataset and the corresponding observed wind speed profiles. The blue curves represent the variation in model-predicted wind speed with altitude, while the red curves denote the wind speed profiles obtained from actual observations.

As indicated by the three sets of wind profile comparisons, the model is able to reproduce the overall vertical variation trends of wind speed across multiple altitude ranges, exhibiting a high degree of consistency, particularly in the lower and upper atmospheric layers. However, more pronounced fluctuations in wind speed are observed at certain specific altitude intervals. These discrepancies may be attributed to more complex atmospheric dynamical processes or localized topographic influences at those heights, which can result in stronger wind variability. Overall, the results suggest that although the current model demonstrates satisfactory overall fitting capability, there remains room for improvement under complex meteorological conditions. Future studies could further enhance the model’s adaptability to complex wind field structures and improve prediction accuracy by incorporating additional meteorological variables or higher-resolution datasets.

## 5. Conclusions

To address the computational complexity and operational cost of traditional physics-based processing in spaceborne Doppler wind lidar applications, this study develops a BP neural network model that learns a nonlinear mapping from Aeolus Rayleigh-channel L1B discriminator responses to the operational L2B horizontal line-of-sight wind (HLOS) product. Using nearly one year of Aeolus data (July 2019–May 2020; 0–20 km), the proposed approach demonstrates good agreement with the L2B reference and reproduces the main statistical characteristics and large-scale HLOS patterns of the L2B product along the satellite tracks. These results indicate that BP neural networks can provide a computationally efficient emulation/approximation of the L2B HLOS output from Rayleigh-channel L1B observables, offering a practical option for fast generation of HLOS estimates in downstream analyses.

Limitations and future work. Since the supervision label and evaluation reference in this study are both the Aeolus L2B product, the reported metrics quantify product-to-product consistency and should not be interpreted as an independently validated improvement in geophysical accuracy. In addition, Aeolus provides single line-of-sight wind components rather than full vector wind direction. Future work will evaluate the model against external or quasi-independent references (e.g., radiosondes or ECMWF analyses) and will report stratified performance by altitude, latitude band, land/ocean, and scene conditions. We will also include additional comparative baselines (e.g., ridge regression, random forest/gradient boosting, and alternative neural architectures) to assess accuracy–cost trade-offs more comprehensively. In addition, we will quantify computational cost by reporting wall-clock timing, throughput, and hardware specifications for both training and inference, to better support near-real-time or quick-look deployment scenarios. Model robustness may be further improved by incorporating additional auxiliary variables and integrating physically informed constraints. Independent validation against external or quasi-independent references is an important next step and is left for future work.

## Figures and Tables

**Table 1:** Input feature inventory (L1B-only) and preprocessing.

Feature	Symbol	Unit	Product Level	Description	Preprocessing	Used as Input
Rayleigh channel signal (channel a)	$R_{a}$	(as in L1B)	L1B	Rayleigh-channel observable from detection channel a	min–max scaling (train-only)	Yes
Rayleigh channel signal (channel b)	$R_{b}$	(as in L1B)	L1B	Rayleigh-channel observable from detection channel b	min–max scaling (train-only)	Yes
Quality/validity flags	–	–	L1B	Used to screen invalid/outlier samples	QC only	No
Target wind (label)	HLOS	m/s	L2B	Horizontal line-of-sight wind	–	Label

**Table 2:** Comparison of Wind Profile Retrieval Transfer Functions.

No.	Hidden-Layer Transfer Function	Output-Layer Transfer Function	Mean Squared Error (MSE)	Coefficient of Determination (*R*^2^)
1	tansig	purelin	0.026298	0.99982
2 3 4	tansig tansig tansig	logsig tansig softmax	955.2784 0.060746 0.059214	−5.5722 0.99958 0.99959
5	purelin	purelin	2.1547	0.98518
6	purelin	logsig	12.286	0.91547
7	purelin	tansig	0.20788	0.99857
8	purelin	softmax	13.8115	0.90498
9	logsig	logsig	0.058742	0.9996
10	logsig	tansig	0.010022	0.99993
11	logsig	purelin	0.005499	0.99996
12	logsig	softmax	0.058387	0.9996

**Table 3:** Train vs. test performance metrics (m/s) against the L2B HLOS reference.

Split	N	Bias (m/s)	MAE (m/s)	RMSE (m/s)	*R* ^2^
Train	8200	−0.030	0.105	0.150	0.99990
Test	660	−0.088	0.128	0.192	0.9998

## Data Availability

The data used in this study are publicly available. The ADM-Aeolus Level-1B (L1B) products analyzed in this work can be obtained from the European Space Agency (ESA) data archive.

## Abbreviations

The following abbreviations are used in this manuscript:

ALADIN	Atmospheric Laser Doppler Instrument
HLOS	Horizontal Line of Sight Wind
ADM-Aeolus	Atmospheric Dynamics Mission
BP	Backpropagation
ICON	Ionospheric Connection Explorer
MIGHTI	Michelson Interferometer for Global High-resolution Thermospheric Imaging
ESA	European Space Agency
DWL	Doppler Wind LiDAR
MOPA	Master Oscillator Power Amplifier
QC	Quality Control
MSE	Mean Squared Error
RMSE	Root Mean Squared Error
MAE	Mean Absolute Error
